# Trends of Hypercholesterolemia Change in Shenzhen, China During 1997–2018

**DOI:** 10.3389/fpubh.2022.887065

**Published:** 2022-05-02

**Authors:** Ke Peng, Weicong Cai, Xiaoying Liu, Yishu Liu, Yu Shi, Jessica Gong, Lin Lei, Ji Peng, Yuxin Xie, Honglei Zhao, Lei Si, Menglu Ouyang

**Affiliations:** ^1^National Clinical Research Center for Cardiovascular Diseases, Fuwai Hospital Chinese Academy of Medical Sciences, Shenzhen, China; ^2^Shenzhen Center for Chronic Disease Control, Shenzhen, China; ^3^The George Institute for Global Health, University of New South Wales, Sydney, NSW, Australia; ^4^School of Medical Science, Faculty of Medicine and Health, University of Sydney, Sydney, NSW, Australia; ^5^School of Public Health, Hengyang Medical School, University of South China, Shenzhen, China; ^6^The George Institute China at Peking University Health Science Center, Beijing, China

**Keywords:** lipids management, hypercholesterolemia, China, cholesterol, epidemiology

## Abstract

To demonstrate the trends of hypercholesterolemia change in Shenzhen, China from 1997 to 2018. Participants were residents aged 18 to 69 years in Shenzhen, China, and were recruited using multi-stage cluster sampling. All participants were surveyed about their socio-demographics, lifestyle, occupation, mental health, and social support. Physical measurements and blood samples for subsequent measurements were collected according to a standardized protocol. A total of 26,621 individuals participated in the three surveys with 8,266 in 1997, 8,599 in 2009, and 9,756 in 2018. In both women and men, there was a significant downward linear trend in age-adjusted mean high-density lipoprotein-cholesterol (HDL-C) from 1997 to 2018 (women: 0.17 ± 0.06, *p* = 0.008 vs. men: 0.21 ± 0.04, *p* < 0.001). In contrast, the age-adjusted total triglycerides and total cholesterol in both sexes have demonstrated an increasing trend in the past two decades. However, no significant changes in age-adjusted low-density lipoprotein-cholesterol (LDL-C) in both men and women between 2009 and 2018 were found (women: 0.00 ± 0.02, *p* = 0.85 vs. men 0.02 ± 0.03, *p* = 0.34). The age-adjusted prevalence of hypercholesterolemia observed a rapid rise from 1997 to 2009 and appeared to be stabilized in 2018, which was similar to the trend of the prevalence of high total triglycerides in women. Changes in trends were varied by different types of lipids traits. Over the observed decades, there was a clear increasing trend of prevalence of low HDL-C (<1.04 mmol/L) in both sexes (women: 8.8% in 1997 and doubled to reach 17.5% in 2018 vs. men was 22.1% in 1997 and increased to 39.1% in 2018), particularly among younger age groups. Hence, a bespoke public health strategy aligned with the characteristics of lipids epidemic considered by sex and age groups needs to be developed and implemented.

## Introduction

Cardiovascular diseases (CVD) are of significant public health concern globally (1). Lipids control is one of the most critical strategies for CVD risk management, as an increased serum level of total cholesterol (TC), low-density lipoprotein cholesterol (LDL-C), and triglycerides (TGs), as well as decreased serum levels of high-density lipoprotein cholesterol (HDL-C) are the major risk factors of CVD (2).

China has witnessed rapid economic growth and development in industry, technology, and urbanization that have significantly altered people's living environments and lifestyles, such as dietary habits, physical activity, and awareness of health and disease (3). Similar to many Western and economically established countries, the general development of the healthcare system in China improved the overall health status and life expectancy, which resulted in rapid population aging. The increased aging population represents a greater number of people at risk of various non-communicable diseases, such as CVDs (4). Moreover, the incidence of stroke and CVD-related risk factors, such as hypertension and dyslipidemia, has increased among the younger population. It has been reported that about 50% of strokes were in patients aged younger than 65 years in 2012 (5). In addition, national-wide surveys revealed that young and middle-aged people lacked the awareness, treatment, and control of hypertension and dyslipidemia (6, 7). As such, China is now confronted with the greatest public health challenge of chronic non-communicable diseases, particularly CVDs (8). According to the data in 2017, stroke and ischemic heart disease have become the leading causes of death in China, resulting in 124–149 deaths per 100,000 people (9). Therefore, it is crucial to monitor CVD-related risk factors and provide appropriate guidance to the at-risk population, policymakers, and health sectors.

Shenzhen is the first Special Economic Zone in China that has become an iconic model of China's rapid development since the 1970s. Shenzhen has approximately 12 million population, 70% being migrant population (10). A cross-sectional study reported that the prevalence of dyslipidemia was 35.7% in the Shenzhen population in 2015 (11). Moreover, the prevalence of CVD risk factors in the population clustering in Shenzhen has been reported to be higher than in other cities in China and also beyond the Chinese national average level (12). A study of a 6-year trend in the serum lipids in Shenzhen has reported a downward trend in LDL-C and an increased trend in low HDL-C (13). However, this study was based on a single local district. There is a lack of CVD risk factors data that encompass a longer time period and include all Shenzhen residents. Therefore, this study aimed to investigate the trends of blood lipids and the prevalence of dyslipidemia among Shenzhen residents, including three population-based cross-sectional studies in 1997, 2009, and 2018.

## Materials and Methods

### Study Design and Participants

Three population-based cross-sectional studies designed to determine the prevalence of non-communicable diseases and risk factors were conducted in Shenzhen in 1997, 2009, and 2018, respectively. A multi-stage stratified cluster random sampling method was used in each of the survey years to select target communities, streets, towns, households, and individuals.

In 1997, one or two street blocks/towns were randomly selected from five administrative districts, including three urban and two rural areas. Subsequently, from each selected street block/town, one or two communities/villages were chosen randomly, for a total of 16 communities/villages. Lastly, residents aged 18–69 years who have lived in Shenzhen for at least half of the past 1 year from each selected household.

In 2009, a probability proportional to size cluster sampling was applied to choose randomly 34 sub-districts from seven administrative districts. Then, a total of 72 communities were randomly chosen from the selected streets, and 120 households were randomly chosen from each of the selected communities. Lastly, a Kish grid was used to select residents aged between 15 and 69 years who have lived in Shenzhen for at least half of the past 1 year from each selected household.

In the study conducted in 2018, 10 communities were first randomly selected from 10 administrative districts, respectively. Subsequently, 100 households were randomly chosen in proportion to their population from each of the selected communities. Lastly, a Kish grid was applied to select one eligible participant who was aged ≥ 18 years and lived in Shenzhen for at least half of the past 1 year from each selected household.

### Data Collection

Face-to-face interviews were conducted to collect basic information about participants, such as sex, age, educational level, marital status, smoking behavior, alcohol use, and physical activity participation, according to a structured questionnaire. Participants aged 18–69 years were included in the analysis. Ethics approval was obtained from the Shenzhen Center for Chronic Disease Control and all participants consented in writing.

The height and weight of the participants were measured based on the standard protocol. Body mass index (BMI) was calculated by dividing weight (kg) by the square of height (m^2^). Overweight was defined as 24.0 ≤ BMI < 28.0 kg/m^2^ and obesity as a BMI of 28.0 kg/m^2^ or above (14–16). Current smokers were defined as participants who have smoked at least one cigarette per day in the past 6 months and currently smoking cigarettes, former smokers were participants who have smoked at least one cigarette each day in the past 6 months and quit smoking for at least 1 month, and never smokers were participants who have never smoked or smoked <1 cigarette in a month. Similarly, current drinkers were defined as participants who have drunk at least one time in a week in the past 6 months and currently consume alcohol, former drinkers as participants who had drank regularly in the past 6 months but quit for at least 1 month, and never drinkers as participants who have never drank or drank <1 time in a month. Participants' involvement in physical activity was measured and categorized into regular physical activity or sedentary, according to whether their participation in the physical activity is at least three times per week and at least 10 min each time.

The participants self-sampled no <30 ml of morning mid urine and sealed and handed it to the study investigators. Overnight fasting blood samples were drawn by venipuncture to measure blood glucose, serum total cholesterol, total triglycerides (TGs), LDL-C, and HDL-C. Blood and urine specimens were collected to measure urine acid, kept at 2–8°C, and sent within 2 h to the Shenzhen laboratory of Guangzhou KingMed Diagnostics Center Co., Ltd, where the specimens were processed and stored at −80°C until laboratory assays could be performed.

Total TGs (≥2.3 mmol/L), total serum cholesterol (≥6.2 mmol/L), LDL-C (≥4.1 mmol/L), and HDL-C (<1.04 mmol/L) cholesterol were classified to be hypercholesterolemia according to the Guidelines for the Prevention and Treatment of Dyslipidemia in Adults in China (2016 Revised Version) (17). Participants were considered to be high uric acid if their uric acid was >420 μmol/L (18) and diabetes mellitus if fasting plasma glucose was ≥ 7.0 mmol/L (19).

### Statistical Analysis

Descriptive statistics were used to summarize the characteristics of the participants by sex and age for each study period. Continuous and categorical variables were expressed as mean (standard deviation, SD) and frequency (percentage), and between-group differences were tested using the *t*-test or one-way ANOVA and χ^2^ test, respectively. Changes in the levels of total TGs, serum total, and HDL cholesterol were determined in comparison with the data in 1997, and changes in LDL-C were determined based on the data in 2009. Restricted maximum-likelihood regressions were used to characterize temporal linear trends. The weights were calculated on the basis of the Chinese Census 2010. SAS analytic software (SAS Institute, Inc) was used for statistical analyses, and a two-tailed *p* < 0.05 was considered statistically significant.

## Results

A total of 26,621 individuals were included in the three surveys with 8,266 in 1997, 8,599 in 2009, and 9,756 in 2018. The proportion of men was 38, 43, and 43%, in 1997, 2009, and 2018, respectively. The characteristics of study populations are presented in Table 1.

**Table 1:** Characteristics of the study population.

	**Man**			**Woman**		
	**1997**	**2009**	**2018**	**1997**	**2009**	**2018**
*N*	3,161	3,736	4,208	5,105	4,863	5,548
*Age, mean ± SD*	37.58 ± 13.05	39.31 ± 11.26	41.90 ± 11.15	37.74 ± 12.55	40.20 ± 12.03	42.79 ± 11.75
*N by age groups (%)*						
18–29	1,011 (31.98)	693 (18.55)	488 (11.60)	1,565 (30.66)	956 (19.66)	626 (11.28)
30–39	933 (29.52)	1,442 (38.60)	1,540 (36.60)	1,534 (30.05)	1,767 (36.34)	1,900 (34.25)
40–49	598 (18.92)	943 (25.24)	1,178 (27.99)	1,068 (20.92)	1,059 (21.78)	1,450 (26.14)
50–59	320 (10.12)	395 (10.57)	597 (14.21)	525 (10.28)	630 (12.95)	891 (16.06)
60–69	299 (9.46)	263 (7.04)	404 (9.60)	413 (8.09)	451 (9.27)	681 (12.27)
*Education, N (%)*						
Illiteracy	109 (3.45)	10 (0.27)	26 (0.62)	499 (9.77)	128 (2.63)	172 (3.10)
Primary school	511 (16.17)	235 (6.29)	260 (6.18)	959 (18.79)	616 (12.67)	764 (13.77)
Middle school	818 (25.89)	1,013 (27.11)	1,102 (26.19)	1,417 (27.76)	1,369 (28.15)	1,535 (27.67)
High school	908 (28.73)	1,230 (32.92)	1,354 (32.18)	1,534 (30.05)	1,614 (33.19)	1,490 (26.86)
College and above	814 (25.76)	1,248 (33.40)	1,466 (34.84)	696 (13.63)	1,136 (23.36)	1,587 (28.60)
*Marital status, N (%)*						
Never married	628 (19.90)	417 (11.16)	373 (8.86)	705 (3.83)	342 (7.03)	305 (5.50)
Married	2,494 (79.05)	3,242 (86.78)	3,742 (88.93)	4,233 (83.03)	4,310 (88.63)	4,960 (89.40)
Divorced	8 (0.25)	37 (0.99)	20 (0.48)	24 (0.47)	101 (2.08)	113 (2.04)
Widowed	18 (0.57)	14 (0.37)	54 (1.28)	131 (2.57)	94 (1.93)	131 (2.36)
Other	7 (0.22)	26 (0.70)	19 (0.45)	5 (0.10)	16 (0.33)	39 (0.70)
*BMI, kg/ m^2^*	22.75 ± 3.31	23.98 ± 3.61	24.22 ± 3.32	22.33 ± 3.41	22.71 ± 3.68	22.93 ± 3.26
*Obesity, N (%)*	207 (6.56)	439 (11.75)	492 (11.69)	316 (6.20)	351 (7.22)	397 (7.16)
*Diabetes, N (%)*	655 (20.72)	213 (5.70)	341 (8.11)	1366 (26.76)	230 (4.73)	352 (6.36)
*High Urine Acid, N (%)*	219 (6.93)	614 (16.43)	867 (20.60)	44 (0.86)	106 (2.18)	234 (4.22)
*Smoking, N (%)*						
Never	1,656 (52.41)	1,761 (47.14)	2,087 (49.60)	5,063 (99.29)	4,771 (98.11)	5,467 (98.54)
Former	200 (6.33)	358 (9.58)	431 (10.24)	12 (0.24)	39 (0.80)	31 (0.56)
Current	1,304 (41.27)	1,617 (43.28)	1,690 (40.16)	24 (0.47)	53 (1.09)	50 (0.90)
*Drinking, N (%)*						
Never Former Current	2,485 (78.64) 49 (1.55) 626 (19.81)	1,897 (50.78) 194 (5.19) 1,645 (44.03)	1,582 (37.75) 302 (7.21) 2,307 (55.05)	5,019 (98.33) 17 (0.33) 68 (1.33)	4,383 (90.13) 66 (1.36) 414 (8.51)	4,240 (76.52) 322 (5.81) 979 (17.67)
*Physical Inactivity, N (%)*						
No	1,085 (34.36)	2,212 (59.27)	1,935 (45.98)	1,241 (24.32)	2,395 (49.27)	1,980 (35.69)
Yes	2,073 (65.64)	1,520 (40.73)	2,273 (54.02)	3,862 (75.68)	2,466 (50.73)	3,568 (64.31)
*Regular physical activity, N (%)*						
No	2,384 (75.49)	2,797 (74.95)	3,052 (72.53)	4,172 (81.76)	3,635 (74.78)	4,129 (74.42)
Yes	774 (24.51)	935 (25.05)	1,156 (27.47)	931 (18.24)	1,226 (25.22)	1,419 (25.58)

### Serum LDL-Cholesterol

The concentrations of serum LDL-C in men and women from 1997 to 2009 are presented in Table 2. The age-adjusted mean LDL-C was 3.15 (SD = 0.78) mmol/L and 3.04 (SD = 0.80) mmol/L for men and women, respectively, in 2009. There were no significant changes of age-adjusted LDL-C in both men and women between 2009 and 2018. For men, LDL-C was the highest among the 50–59-year-olds in both 2009 and 2018. For women, the 60–69-year-olds constantly showed the highest LDL-C among all age groups across time. The concentrations of LDL-C increased with age in both sexes. The age group 40–49 years achieved the largest decrease (−0.11 ± 0.03 mmol/L) in LDL-C among men, and the 60–69 years achieved the largest drop (−0.15 ± 0.05 mmol/L) among women.

**Table 2:** Age-adjusted lipid profiles from 1997 to 2018.

	**Man**				**Woman**			
	**1997**	**2009**	**2018**	***P* for difference^**†**^**	**1997**	**2009**	**2018**	***P* for difference**
LDL-C, mean (mmol/L) ± SD								
18–29	NA	2.82 ± 0.76	0.13 ± 0.06	0.004	NA	2.59 ± 0.67	0.07 ± 0.04	0.038
30–39	NA	3.08 ± 0.74	0.01 ± 0.03	0.669	NA	2.77 ± 0.66	0.01 ± 0.01	0.594
40–49	NA	3.26 ± 0.78	−0.11 ± 0.03	0.002	NA	3.04 ± 0.71	−0.04 ± 0.03	0.192
50–59	NA	3.28 ± 0.72	0.01 ± 0.05	0.852	NA	3.40 ± 0.81	−0.07 ± 0.04	0.103
60–69	NA	3.18 ± 0.75	−0.03 ± 0.03	0.637	NA	3.51 ± 0.85	−0.15 ± 0.05	0.003
Age-adjusted mean		3.15 ± 0.78	0.00 ± 0.02	0.850		3.04 ± 0.80	−0.02 ± 0.03	0.335
HDL-C, mean (mmol/L) ± SD		P for LT^*^				P for LT
18–29	1.32 ± 0.32	−0.14 ± 0.05	−0.20 ± 0.10	<0.001	1.53 ± 0.34	−0.21 ± 0.07	−0.26 ± 0.06	<0.001
30–39	1.27 ± 0.32	−0.15 ± 0.05	−0.17 ± 0.05	<0.001	1.49 ± 0.35	−0.17 ± 0.05	−0.23 ± 0.04	<0.001
40–49	1.25 ± 0.31	−0.13 ± 0.10	−0.15 ± 0.05	<0.001	1.45 ± 0.35	−0.15 ± 0.06	−0.19 ± 0.05	<0.001
50–59	1.29 ± 0.33	−0.17 ± 0.10	−0.19 ± 0.10	<0.001	1.44 ± 0.36	−0.14 ± 0.10	−0.17 ± 0.08	<0.001
60–69	1.33 ± 0.36	−0.21 ± 0.08	−0.18 ± 0.07	<0.001	1.40 ± 0.35	−0.09 ± 0.06	−0.15 ± 0.10	<0.001
Age-adjusted mean	1.29 ± 0.33	−0.16 ± 0.07	−0.17 ± 0.06	0.008	1.47 ± 0.35	−0.16 ± 0.05	−0.21 ± 0.04	<0.001
Total cholesterol, mean (mmol/L) ± SD								
18–29	4.34 ± 0.89	0.25 ± 0.07	0.34 ± 0.11	<0.001	4.28 ± 0.86	0.11 ± 0.02	0.13 ± 0.02	0.001
30–39	4.62 ± 1.07	0.29 ± 0.08	0.25 ± 0.09	<0.001	4.43 ± 0.86	0.18 ± 0.03	0.09 ± 0.02	0.007
40–49	4.81 ± 1.11	0.33 ± 0.09	0.16 ± 0.08	0.054	4.79 ± 1.01	0.18 ± 0.08	0.04 ± 0.04	0.502
50–59	4.93 ± 1.10	0.19 ± 0.08	0.17 ± 0.07	0.038	5.23 ± 1.04	0.18 ± 0.03	0.05 ± 0.05	0.684
60–69	4.88 ± 0.96	0.11 ± 0.07	0.03 ± 0.01	0.786	5.30 ± 1.07	0.28 ± 0.05	0.01 ± 0.03	0.444
Age-adjusted mean	4.70 ± 1.05	0.28 ± 0.08	0.24 ± 0.07	0.005	4.74 ± 1.03	0.22 ± 0.06	0.06 ± 0.03	<0.001
Total triglycerides, mean (mmol/L) ± SD								
18–29	1.04 ± 0.82	0.41 ± 0.12	0.48 ± 0.10	<0.001	0.78 ± 0.54	0.24 ± 0.08	0.19 ± 0.09	<0.001
30–39	1.41 ± 1.50	0.57 ± 0.16	0.35 ± 0.08	<0.001	0.95 ± 0.84	0.18 ± 0.07	0.10 ± 0.06	0.001
40–49	1.57 ± 1.67	0.58 ± 0.11	0.37 ± 0.09	0.004	1.17 ± 1.12	0.29 ± 0.07	0.05 ± 0.05	0.594
50–59	1.53 ± 2.29	0.40 ± 0.14	0.25 ± 0.12	0.090	1.44 ± 1.09	0.35 ± 0.09	0.09 ± 0.06	0.594
60–69	1.26 ± 0.84	0.45 ± 0.17	0.14 ± 0.04	0.187	1.58 ± 1.50	0.31 ± 0.07	−0.10 ± 0.09	0.085
Age-adjusted mean	1.37 ± 1.52	0.56 ± 0.15	0.42 ± 0.10	<0.001	1.13 ± 1.06	0.30 ± 0.08	0.14 ± 0.07	<0.001

*Data for low-density lipoprotein cholesterol (LDL-C) were not available in 1997*.

† *The value of p for difference between 2009 and 2018*.

* *The value of p-value for linear trend across years*.

### Serum HDL-Cholesterol

The age-adjusted mean serum HDL-C concentration in 1997 was 1.29 (SD = 0.33) mmol/L in men and 1.47 (SD = 0.35) mmol/L in women (Table 2). In both sexes, there was a significant downward linear trend in age-adjusted mean HDL-C from 1997 to 2018. Women had a higher age-adjusted mean HDL-C in 1997 than men. However, a larger drop in magnitude (−0.21 vs. −0.17 mmol/L) was also observed among women in 2018. From 1997 to 2018, significant decreasing trends of HDL-C were observed for all age groups in both sexes. For men, those aged 60–69 years showed the highest HDL-C in 1997, however, they also demonstrated the largest decrease over the years. By 2018, HDL-C was the highest in 18–29-year-oldmen. Women aged 18–29 years had the highest HDL-C in 1997 but had the largest decrease over the years. The 60–69-year-old women with the lowest HDL-C in 1997 showed the smallest reduction in 2018.

### Serum Total Cholesterol

The age-adjusted total cholesterol concentration was 4.70 (SD = 1.05) mmol/L in men and 4.74 (SD = 1.03) mmol/L in women in 1997 (Table 2). There was an increasing trend of age-adjusted mean total cholesterol in both sexes across years. The younger groups of men and women aged 18–39 with lower total cholesterol levels in 1997 showed larger increases than other age groups over the years. For 40–49-year-olds in men in 2009, the total cholesterol had a sharp increase by 0.33 (SD = 0.09) mmol/L compared with 1997, reaching the borderline increase of total cholesterol defined by the guideline (2016 version). The older age groups (50–69 years) had a higher concentration of total cholesterol in 1997 and it remained stable over the years among both men and women. The 50–69 years groups in women showed total cholesterols above 5.2 mmol/L, exceeding the cut-off of borderline increase in 1997 and continuing to rise by small magnitudes in 2009 and 2018.

### Serum Total Triglycerides

In 1997, the age-adjusted total TGs concentration was higher in men (1.37 mmol/L, SD = 1.52) than in women (1.13 mmol/L, SD = 1.06) (Table 2). The age-adjusted total TGs in both sexes demonstrated an increasing trend in the past two decades. The younger groups aged below 40 years, for both men and women, showed a larger and more rapid rise in total TGs. For men, total TGs were the highest in the 40–59 age groups with large increases in 2009 and smaller increases in 2018. The women aged 40–69 years experienced a larger rise in total TGs than other age groups in 2009 and the concentrations remained stable from 2009 to 2018.

### Prevalence of Hypercholesterolemia

**Figure 1:**
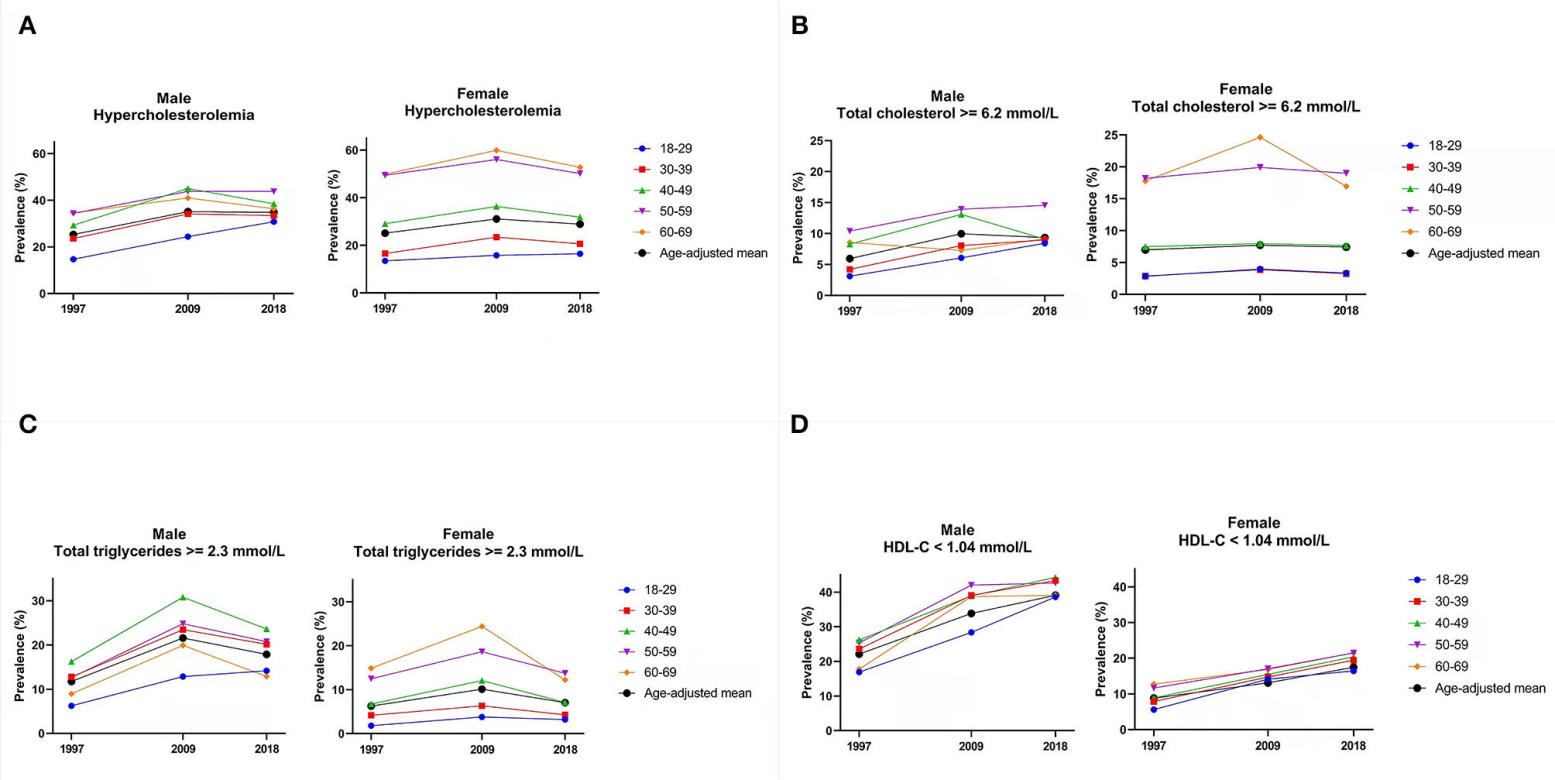
Age-adjusted prevalence of lipid profiles trends from 1997 to 2018. Footnote: Hypercholesterolemia is defined as total triglycerides (≥2.3 mmol/L), total serum cholesterol (≥6.2 mmol/L), low-density lipoprotein cholesterol (LDL-C) (≥4.1 mmol/L), and high-density lipoprotein cholesterol (HDL-C) (<1.04 mmol/L). **(A)** Prevalence of hypercholesterolemia. **(B)** Prevalence of high total cholesterol. **(C)** Prevalence of high total triglycerides. **(D)** Prevalence of low HDL-cholesterol.

The age-adjusted prevalence of hypercholesterolemia in both men and women showed a rapid rise from 1997 to 2009 and stabilized in 2018 (Figure 1A). The age-adjusted prevalence was similar in both sexes in 1997 (about 25%) and rose to 34.8% among men in 2018. Whereas, in 2018, the prevalence among women decreased to 28.9%. The prevalence of hypercholesterolemia among men aged below 50 years was consistently higher than that of women of the same age. At older ages (50–69 years), hypercholesterolemia was more prevalent in women than in men. Among women, the prevalence of hypercholesterolemia increased with age. This pattern was not observed in men aged over 40 years among which the prevalence was similar across older ages.

### Prevalence of High Total Cholesterol

The age-adjusted prevalence of increased total cholesterol (≥6.2 mmol/L) was 5.9% in 1997, increased to 9.96% in 2009, and then slightly decreased to 9.3% in 2018 among men (Figure 1B). The age-adjusted prevalence among women was 7.0% and remained stable over the years. The prevalence was similar between the two youngest groups of women and increased with age. This pattern was also observed among men except for the 60–69 years among which the prevalence was lower than the 40–49 years and 50–59 years, and comparable with the age-adjusted mean prevalence.

### Prevalence of High Total TGs

The prevalence of high total triglycerides among women increased with age and was consistently lower than that of men (Figure 1C). For some age groups of women, the prevalence of high total TGs rose in 2009 and either stabilized or decreased in 2018. For men, the prevalence of high total triglycerides was the highest among those aged 40–49 years. The prevalence among the 30–39 and 50–59 years was similar. The 60 to 69 years age group showed the second-lowest prevalence to high total triglycerides, only higher than the youngest age group.

### Prevalence of low HDL-Cholesterol

The prevalence of low HDL-C among women was lower than that of men, and there were no large disparities between age groups (Figure 1D). There was a clear increasing trend in both sexes across years. The age-adjusted prevalence in women was 8.8% in 1997 and doubled to reach 17.5% in 2018. The age-adjusted prevalence in men was 22.1% in 1997 and increased to 39.1% in 2018. The prevalence among men was similar across the age groups.

## Discussion

In this large population-based cohort in Shenzhen over the past two decades, the serum LDL-C has significantly increased from 2007 to 2018 in those of younger age, especially in men (increased 0.13 ± 0.06, *p* = 0.004 in age group of 18–29 years). In contrast, there was a significant decrease in HDL-C in both sexes but more prone in women of younger age (men: −0.20 ± 0.10 vs. women: −0.26 ± 0.06 in age group 18–29 years). The total cholesterol increased in both sexes from 1997 to 2018, but men had a higher increase than women (men: 0.24 ± 0.07 vs. women: 0.06 ± 0.03 from 2007 to 2018). A similar trend was observed in total triglycerides. The prevalence of hypercholesterol increased from 1997 to 2009 but decreased slightly from 2009 to 2018, while the prevalence of low HDL-C increased in both women and men over the decades. In this study, we found the increases in LDL-C and total cholesterol in lipidemia profile were significant at a younger age compared with the older population. A previous study observed the highest estimate of dyslipidemia prevalence (49.3%) in people over 30 years of age in the eastern region of China (20). A large population-based study reported that work-related stress was associated with high LDL-C, low HDL-C, total cholesterol, and hypercholesterolemia diagnosis (21). Shenzhen is a highly developed and urbanized city, with a stressful lifestyle contributing to increasing work pressure, decreased physical activity, and unhealthy diet, particularly among the young to middle-aged population, with men being more likely than women to have increased total cholesterol. This is consistent with the findings from a review on dyslipidemia epidemiology in the Chinese population (20). A possible explanation for this sex disparity in dyslipidemia could be that there was a higher proportion of obesity, cigarette smoking, and alcohol consumption in men compared with women. This may also be relevant to sex differences in awareness, treatment, and self-management. A previous study found that women were more aware of dyslipidemia, more likely to receive treatments for it, and more likely to have their condition controlled compared with men (22). However, the higher prevalence of hypercholesterolemia and low HDL-C in women than in men in the age group of 40–59 years should be noted. This may be related to sex hormones changes that affect the lipidemia parameters due to menopause in women (23).

The prevalence of low HDL-C (42.5%) in 2018 in this study was higher than in the previous studies [30.4% (6) and 41.9% (21)] among Chinese adults. The greater burden of dietary-related chronic conditions, such as dyslipidemia, has been reported in economically developed and highly urbanized areas, predominantly in the east coast of China (24). This may be related to the fact that highly economically developed areas are more likely to have better healthcare services and accessibility to healthcare facilities, where more people can access prompt diagnosis and, therefore, contributed to the higher reported prevalence of dyslipidemia among populations in Shenzhen.

We found that the prevalence of hypercholesterol was decreased in older age people during the most recent decade (2008–2018), which might be related to the implementation of health policy and advocacy in public health strategies for lipid control in the older people in recent years (25). Though age has been reported as the most devastating contributor of dyslipidemia, implementation of policies, such as increase the accessibility of diagnostic technologies, improving awareness of risk factors, changing healthy lifestyles to prevent CVDs, and developing effective hypolipidemic medications, is beneficial for long-term outcomes in the older population (26). In 2004, the China cholesterol education program was initiated for improving and standardizing the interventions for hyperlipidemia management (27). Moreover, since 2007, a joint committee of multidisciplinary experts formulated the “Chinese guidelines for the management of dyslipidemia in adults,” which has provided guidance and strategies to effectively control dyslipidemia from a clinical perspective (28). Although a downward trend in the prevalence of dyslipidemia was observed in the study population, other factors associated with the risk of cardiovascular disease, such as hypertension, diabetes mellitus, and lifestyle factors, must be considered when developing prevention strategies. Additionally, total and LDL-C levels tend to increase with age in young or middle-aged adults when evaluated cross-sectionally or prospectively.

### Strengths and Weaknesses

The major strength of the current study was the use of a purposeful sampling strategy in a large and representative population, which reduced the risk of selection bias with good generalizability. However, there are several limitations: this is a cross-sectional study rather than a prospective cohort, therefore, no causal relationships can be determined and the data collected, such as former smokers/drinkers might not be long enough to show a difference in the blood lipid level. Another limitation is the potential selection bias that is induced by emigration and immigration in Shenzhen City. Because of how fast residents change during these two decades, the random selected households might not be representative compared with the others living in the same community. The prevalence of dyslipidemia differences is also relevant to differences in awareness, treatment, and self-management between sexes and age groups. However, this information was not collected, which may influence the interpretation of the findings. Moreover, even though blood sample tests were standardized in all the surveys, measurement errors could not be avoided.

## Conclusion

In conclusion, the current study investigated the trends of different types of lipid abnormalities in Shenzhen city, China-based on large-scale population samples. The age-adjusted prevalence of hypercholesterolemia observed a rapid rise from 1997 to 2009 and appeared to be stabilized in 2018. There was a clear increasing trend in the prevalence of low HDL-C (<1.04 mmol/L) in both sexes over the observed decades. To improve lipids management in Shenzhen city, it is crucial to explore the underlying reasons for the change in trends, to allow for appropriate public health strategies to be developed and implemented in Shenzhen.

## Data Availability Statement

The original contributions presented in the study are included in the article/supplementary materials, further inquiries can be directed to the corresponding authors.

## Ethics Statement

The studies involving human participants were reviewed and approved by Shenzhen Center for Chronic Disease Control. The patients/participants provided their written informed consent to participate in this study.

## Author Contributions

KP, LL, and JP conceptualized the study. KP, XL, YX, and MO contributed to methodology. WC and YL contributed to formal analysis, software, and analysis. YL, MO, and XL validated the study. LL contributed to resources. MO, XL, YL, WC, YS, and KP wrote the original draft. LS, JG, HZ, and KP contributed to writing, reviewing, and editing the manuscript. YL visualized the study. LL, JP, and KP contributed to project administration. All authors have read and agreed to the published version of the manuscript.

## Funding

This research is supported by the Sanming Project of Medicine in Shenzhen [Grant Number: SZSM201911015], the Guangdong Basic and Applied Basic Research Foundation (2019A1515111003 and 2021A1515110307), and the Young Talent Program of the Academician Fund, Fuwai Hospital Chinese Academy of Medical Sciences, Shenzhen YS-2020-006. LS is supported by a National Health and Medical Research Council Early Career Fellowship [Grant Number: GNT1139826].

## Conflict of Interest

The authors declare that the research was conducted in the absence of any commercial or financial relationships that could be construed as a potential conflict of interest.

## Publisher's Note

All claims expressed in this article are solely those of the authors and do not necessarily represent those of their affiliated organizations, or those of the publisher, the editors and the reviewers. Any product that may be evaluated in this article, or claim that may be made by its manufacturer, is not guaranteed or endorsed by the publisher.
